# Direct interaction of Ikaros and Foxp1 modulates expression of the G protein-coupled receptor G2A in B-lymphocytes and acute lymphoblastic leukemia

**DOI:** 10.18632/oncotarget.11688

**Published:** 2016-08-30

**Authors:** Jonathan Bond, Renae Domaschenz, Mónica Roman-Trufero, Pierangela Sabbattini, Isabel Ferreiros-Vidal, Gareth Gerrard, Vahid Asnafi, Elizabeth Macintyre, Matthias Merkenschlager, Niall Dillon

**Affiliations:** ^1^ Gene Regulation and Chromatin Group, MRC Clinical Sciences Centre, Imperial College Faculty of Medicine, Hammersmith Campus, London W12 0NN, United Kingdom; ^2^ Lymphocyte Development Group, MRC Clinical Sciences Centre, Imperial College Faculty of Medicine, Hammersmith Campus, London W12 0NN, United Kingdom; ^3^ Imperial Molecular Pathology, Imperial College Academic Health Sciences Centre, Hammersmith Campus, London W12 0NN, United Kingdom; ^4^ Université Paris Descartes Sorbonne Cité, Institut Necker-Enfants Malades (INEM), Institut National de Rrecherche Médicale (INSERM), and Laboratory of Onco-Hematology, Assistance Publique-Hôpitaux de Paris (AP-HP), Hôpital Necker Enfants-Malades, Paris, France; ^5^ Present address: Chromatin and Transcriptional Regulation Group, John Curtin School of Medical Research, The Australian National University, Canberra, Australian Capital Territory, Australia

**Keywords:** Ikaros, Foxp1, GPR132, B cell cycle, acute leukemia

## Abstract

Ikaros and Foxp1 are transcription factors that play key roles in normal lymphopoiesis and lymphoid malignancies. We describe a novel physical and functional interaction between the proteins, which requires the central zinc finger domain of Ikaros. The Ikaros-Foxp1 interaction is abolished by deletion of this region, which corresponds to the IK6 isoform that is commonly associated with high-risk acute lymphoblastic leukemia (ALL). We also identify the *Gpr132* gene, which encodes the orphan G protein-coupled receptor G2A, as a novel target for Foxp1. Increased expression of Foxp1 enhanced *Gpr132* transcription and caused cell cycle changes, including G2 arrest. Co-expression of wild-type Ikaros, but not IK6, displaced Foxp1 binding from the *Gpr132* gene, reversed the increase in *Gpr132* expression and inhibited G2 arrest. Analysis of primary ALL samples revealed a significant increase in *GPR132* expression in *IKZF1*-deleted BCR-ABL negative patients, suggesting that levels of wild-type Ikaros may influence the regulation of G2A in B-ALL. Our results reveal a novel effect of Ikaros haploinsufficiency on Foxp1 functioning, and identify G2A as a potential modulator of the cell cycle in Ikaros-deleted B-ALL.

## INTRODUCTION

Progression through B cell differentiation is tightly regulated by a specific transcription factor program in which members of the Ikaros and forkhead families have key roles. Ikaros is a Kruppel-like factor [1, 2] whose expression has been shown to be absolutely required for B cell development [3]. Mice that are null for Ikaros exhibit a block in B cell differentiation at a very early stage of the differentiation process, before the cells reach the pro-B cell stage [3], whereas mice that have a conditional Ikaros deletion at the pro-B cell stage show a block in pro-B to pre-B cell differentiation [4, 5]. Ikaros plays essential roles in regulating cell cycle exit during the transition from the large cycling pre-B to the small resting pre-B cell stage by downregulating expression of the pre-B cell receptor, which stimulates pre-B cell proliferation [6–8]. Genome-wide analysis of Ikaros binding to gene promoters has also revealed occupancy of a wide-range of promoters of genes that are involved in regulation of B cell differentiation [9].

Mutations and deletions in the *IKZF1* gene, which encodes Ikaros, are particularly associated with Philadelphia positive (Ph^+^, also known as BCR-ABL positive) B-ALL and are present in 70-80% of these patients. In the majority of these cases, Ikaros haploinsufficiency is caused by complete or partial gene deletion, which frequently results in expression of a dominant negative IK6 isoform that lacks the central zinc finger DNA-binding domain [10–12]. Several studies have shown *IKZF1* deletions to be associated with poor outcome in both Ph+ and Ph- B-ALL, suggesting that Ikaros haploinsufficiency is likely to contribute directly to poor treatment response in these patients [13–15].

Foxp1 is a member of the forkhead family of transcription factors, which has been shown to be required for B cell development [16, 17]. Foxp1 null mice have a block on B cell differentiation at the pro-B cell stage and Foxp1 has been implicated as an activator of the Erag enhancer, which controls expression of the *Rag* genes [16]. Foxp1 has also been implicated in pre-B cell differentiation and control of mature B cell numbers. Knockdown of the microRNA miR-34a increases expression of Foxp1 in pre-B cells and results in increased numbers of mature B cells, while leaving pro- and pre-B cell numbers unaffected [18]. This result has led to the suggestion that the level of Foxp1 influences the rate of differentiation of pre-B cells into immature and mature B cells [18].

The *Gpr132* gene encodes the G2A protein, which was originally identified as an orphan G protein-coupled receptor (GPCR) that is induced by DNA damage and stress and blocks cells in G2/M [19]. Mice that are null for the *Gpr132* gene exhibit a severe late-onset autoimmune syndrome, which is characterised by abnormal expansion of both T and B lymphocytes [20]. The G2A protein has been shown to act as a tumour suppressor in mouse pre-B cells where it antagonises the effect of BCR-ABL [21]. However, it also has oncogenic properties when expressed in NIH3T3 fibroblasts [22]. These results indicate that *Gpr132* expression has different effects on the cell cycle and proliferation depending on context and suggest that varying levels of expression of the gene could influence the behaviour of cancers in complex ways.

In this study, we identify a novel interaction between Ikaros and Foxp1 in pre-B cells and B-ALL cells, which is abolished by the IK6 deletion. We also show that *Gpr132* is a target for activation by Foxp1 through direct binding to the gene. Overexpression of Foxp1 in pre-B cells results in increased *Gpr132* transcription and significant effects on the cell cycle, including G2 arrest. Co-expression of wild-type Ikaros, but not the IK6 deletion mutant, antagonises the enhancing effects of Foxp1 on *Gpr132* transcription and blocks the G2 arrest phenotype. We also show that *GPR132* levels are significantly increased in BCR-ABL-negative B-ALL patients that have the IK6 deletion. Our results provide evidence of an interplay between two key regulators of the cell cycle in B-ALL and suggest that *GPR132* expression could be a parameter that influences cell cycle behaviour and outcome in these patients.

## RESULTS

### Ikaros and Foxp1 interact in vitro and in vivo

**Figure 1:**
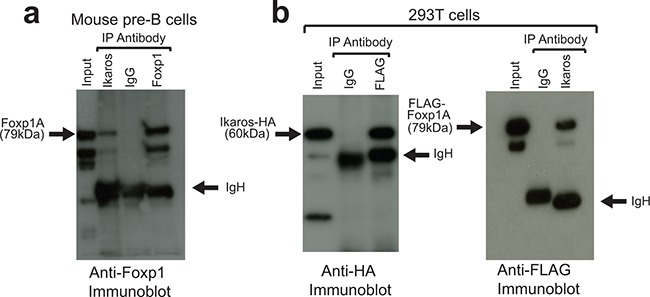
Ikaros and Foxp1 proteins interact in vivo **A.** Protein extracts from murine fetal liver pre-B-cells were subjected to IP with anti-Foxp1 and anti-Ikaros antibodies and with control Ig and immunoblotted with anti-Foxp1. Arrows indicate full-length Foxp1 (Foxp1A) and IgH. The higher mobility bands are likely to be shorter Foxp1 splicing isoforms that are precipitated by the anti-Ikaros and anti-Foxp1 antibodies. **B.** IPs were carried out on protein lysates from 293T cells following co-transfection with FLAG-tagged Foxp1 and HA-tagged Ikaros constructs. Left panel: IP with anti-FLAG (Foxp1); immunoblotting with anti-HA (Ikaros). Right panel: IP with anti-Ikaros; immunoblotting with anti-FLAG (Foxp1). IP was carried out with anti-Ikaros antibody because the tagged Ikaros was only weakly precipitated from extracts by anti-HA, most likely due to epitope occlusion. Input for (A) and (B) = 5% of amount of protein lysate used for IPs.

The possibility that Ikaros and Foxp1 interact directly or as part of a multi-protein complex in pre-B cells was tested by co-immunoprecipitation (co-IP) of protein lysates obtained from wild-type murine fetal liver pre-B cells with anti-Ikaros and anti-Foxp1 antibodies. IP of a pre-B cell lysate with anti-Ikaros antibody showed a significant pulldown of Foxp1 (Figure 1A). In order to confirm the interaction further, constructs that encoded HA-tagged Ikaros and FLAG-tagged Foxp1 were co-transfected into 293T cells. Reciprocal pulldowns with anti-FLAG and anti-Ikaros demonstrated that the tagged proteins interact strongly in 293T cells (Figure 1B). Treatment of the extracts with DNase I had no effect on the pulldowns (Supplementary Figure S1A) and a non-DNA-binding Ikaros 159A mutant [23] was also shown to interact with Foxp1 in pulldown assays in 293T cells (Supplementary Figure S1B). These results demonstrate that the interaction was not dependent on the two factors binding simultaneously to the same DNA region.

**Figure 2:**
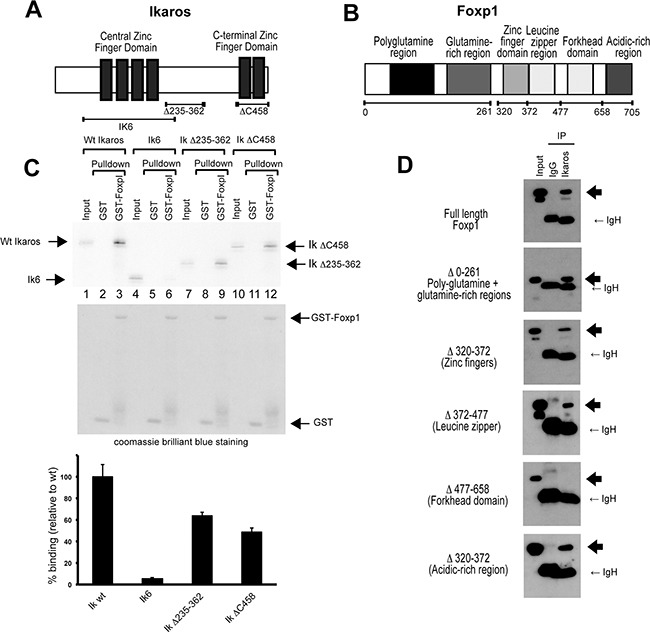
Identification of the interaction domains in Ikaros and Foxp1 **A.** Schematic representation of the zinc-finger domains of mouse Ikaros. **B.** Schematic representation of the functional domains of mouse Foxp1. **C.** Mapping of the Foxp1 interaction domain in the Ikaros protein by GST pulldown (see Methods). The upper panel shows pulldowns of S35- labelled Ikaros-deletion proteins with GST (lanes 2, 5, 8 and 11) or with GST-Foxp1 (lanes 3, 6, 9 and 12). Ikaros deletions are shown schematically in (A). The middle panel shows the coomassie-stained gel confirming the presence of GST and GST-Foxp1. The lower panel shows the results of the quantification of the pulldown bands in the upper panel. Values are represented as percentage binding relative to the wild-type value, which was set at 100%. The results shown are the mean of 3 experiments. Values for each pulldown were calculated relative to input. The relative pulldown values for wild-type Ikaros were then set at 100% for each experiment, and the value for each deletion was calculated as a percentage of the wild-type value for that experiment. The mean percentage pulldown and SD for each construct was then calculated. **D.** Interaction domains in the Foxp1 protein were mapped by co-transfection of 293T cells with WT Ikaros and Foxp1 deletion mutants that lack specific structural domains (depicted in (B)). Heavy arrows indicate the positions of the Foxp1 deletion proteins on the western blots.

The Ikaros and Foxp1 proteins contain multiple domains that are involved in mediating their DNA binding and protein-protein interaction functions (Figure 2A and 2B). To identify regions that are involved in the interaction between the two proteins, a series of deletions was generated in both proteins and the effect on the Ikaros-Foxp1 interaction was tested using an *in vitro* GST pulldown assay and by co-expression of the proteins in 293T cells (Figure 2C and 2D). *In vitro* pulldown using bacterially expressed GST-Foxp1 fusion protein and ^35^S-labelled Ikaros generated by *in vitro* translation demonstrated that Ikaros and Foxp1 interact directly (Figure 2C, lanes 1-3 WT Ikaros). The Ikaros protein contains a central zinc finger domain (Figure 2A) that comprises four zinc fingers and is responsible for DNA binding specificity. The C-terminal region of the protein contains two zinc fingers and has been shown to mediate protein-protein interactions [24]. Deletion of exons 3-6, which encode the four central zinc fingers, created a truncated protein that is equivalent to the human IK6 mutant (Figure 2A). The IKΔC458 deletion removed the two C-terminal zinc fingers that mediate the formation of homo- and heterodimers, whereas the IKΔ235-362 deletion removed a major part of the region between the two zinc finger domains. Analysis of the effect of the deletions on the efficiency of pulldown by GST-Foxp1 showed that the IK6 deletion reduced binding to 2% of the level observed for the wild-type protein (Figure 2C, lanes 4-6). The IKΔ235-262 and IKΔC458 deletions gave reductions of 40% and 50% of wild-type binding levels respectively (Figure 2C, lanes 7-12). This suggests minor additional contributions from these regions to the interaction, which could be due to effects on the 3-dimensional structure of the protein. Our results indicate that the DNA binding region of Ikaros has a major role in the interaction with Foxp1 and show that deletion of this region is sufficient to abrogate the interaction.

The interaction domains in the Foxp1 protein were analysed by co-expressing FLAG-tagged Foxp1 deletion mutants with wild-type Ikaros in 293T cells (Figure 2B and 2D). All of the Foxp1 deletion mutants interacted efficiently with Ikaros with the exception of a deletion that removed the forkhead domain (Δ477-658), which is known to mediate binding to its consensus DNA recognition sequence [25]. Overall, the data from the deletion analysis demonstrate that the main interaction domains are located in the zinc finger DNA binding region of Ikaros and the forkhead DNA binding region of Foxp1.

### The human Ikaros and FOXP1 proteins interact in B-ALL cells and this interaction is abolished by the IK6 deletion

**Figure 3:**
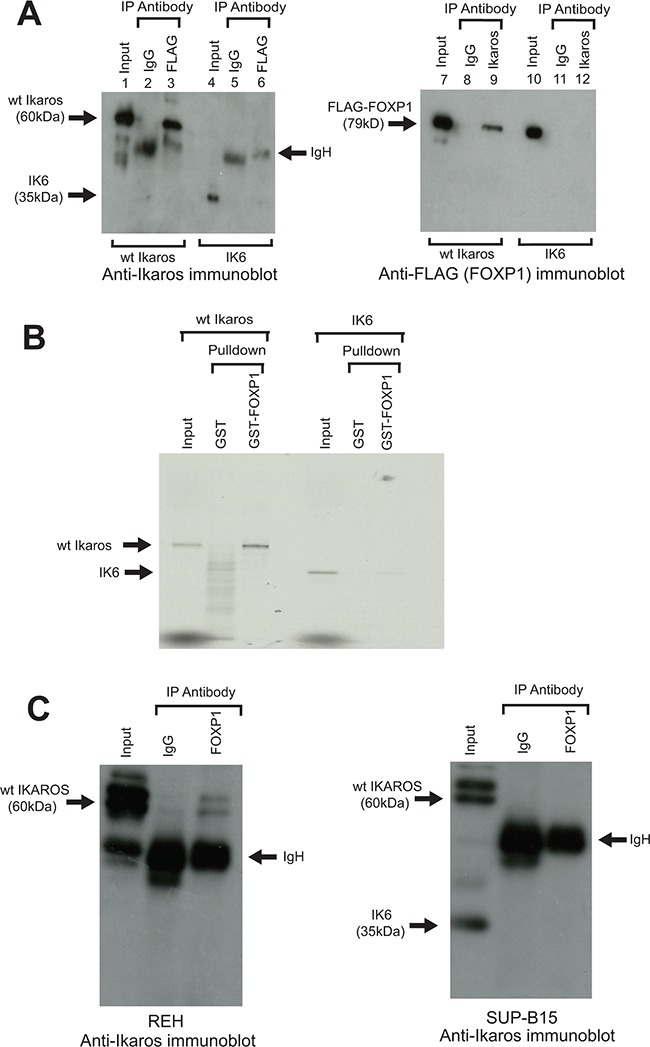
Interaction of human Ikaros and FOXP1 in B-ALL cells and effect of the IK6 deletion **A.** A FLAG-tagged human FOXP1 construct was co-transfected into 293T cells with wild-type (wt) human Ikaros constructs (lanes 1 - 3 and 7 - 9), or human IK6 (lanes 4 - 6 and 10 - 12). Following IP with anti-FLAG or anti-Ikaros, samples were immunoblotted with anti-Ikaros (left panel) or anti-FLAG (FOXP1) (right panel). **B.** GST pulldown assay using a human GST-FOXP1 fusion and ^35^S labelled wt human Ikaros or the human IK6 isoform. **C.** Anti-Foxp1 IP of Ikaros isoforms in human leukemia cell lines. The REH line (left panel) expresses WT Ikaros. The SUP-B15 line (right panel) expresses wt Ikaros and the IK6 deletion isoform. Arrows indicate wt Ikaros, IK6, FLAG-Foxp1 and IgH on the gels.

The human Ikaros and FOXP1 proteins show greater than 90% homology with their counterparts in the mouse, suggesting that the human proteins are also likely to interact. To test whether this is the case, tagged human Ikaros and FOXP1 proteins were initially co-expressed in 293T cells. Reciprocal pulldown of both proteins in extracts from the transfected cells using antibodies that recognise the tags provided a clear demonstration that they interact (Figure 3A). When the cells were co-transfected with human IK6 and FLAG-tagged FOXP1 expression constructs, IP with anti-FLAG antibody showed that the IK6 protein does not co-IP with FOXP1 in this system (Figure 3A). *In vitro* pulldown with the GST-FOXP1 fusion protein showed that pulldown of human IK6 was drastically reduced compared with the pulldown observed with wild-type human Ikaros (Figure 3B).

To determine whether the interaction between Ikaros and FOXP1 occurs in the context of human B-ALL, co-IP analysis was carried out using nuclear extracts from REH and SUP-B15 human B-ALL cell lines. REH cells express wild-type Ikaros. The SUP-B15 line has a heterozygous *IKZF1* deletion and expresses both the IK6 isoform and wild-type Ikaros (Figure 3C). Co-IP of extract from REH cells with anti-FOXP1 resulted in pulldown of wild-type Ikaros (Figure 3C, left panel). In contrast, the co-IP of SUP-B15 extract with anti-FOXP1 did not detect pulldown of the IK6 isoform, although we cannot exclude the possibility that there is some interaction that is below the detection level of the blot (Figure 3C, right panel). Together with the data from the *in vitro* pulldown with GST-FOXP1, these results provide strong evidence that that the IK6 deletion disrupts the Ikaros-FOXP1 interaction in B-ALL cells. Co-IP with the anti-FOXP1 antibody also gave much less pulldown of wild-type Ikaros from SUP-B15 lysates compared to REH cells. The apparent reduction in the interaction of FOXP1 with wild-type Ikaros could be partly due to the reduced levels of wild-type Ikaros in the SUP-B15 line (see Figure 3C, right panel, input lane), but it could also be caused by sequestration of wild-type Ikaros by IK6 [24]. It should also be noted that previous studies have shown that uptake of IK6 into the nucleus has been shown to be significantly reduced compared with wild-type Ikaros [26]. However, in cells that express both wild-type and IK6 isoforms, as is the case in the SUP-B15 cell line, dimerization between wild-type Ikaros and IK6 has been shown to result in nuclear localisation of IK6 [24]. These results suggest that failure of IK6 to interact with FOXP1 in the nucleus in B-ALL cells could be partly due to a direct effect on Ikaros-FOXP1 interaction, as well as being a function of cytoplasmic localisation of the IK6 isoform. The overall effect on the functioning of FOXP1 would be similar to the block on direct interaction demonstrated in Figure 3B.

### Foxp1 enhances expression of Gpr132

**Figure 4:**
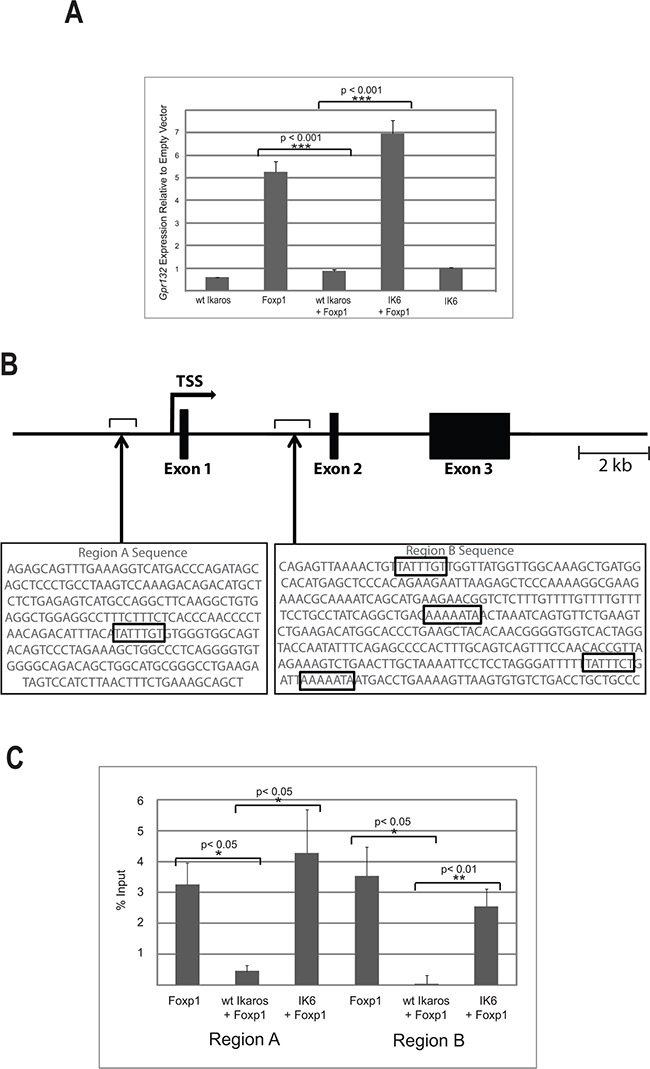
Ikaros antagonises the activating effect of Foxp1 on Gpr132 expression **A.** qRT-PCR analysis of *Gpr132* expression in B3 cells expressing ectopic wild-type (wt) Ikaros, IK6 and Foxp1. *Gpr132* levels are expressed relative to cells transduced with empty vector control, therefore levels > 1 indicate increased expression. Values are the mean ± SEM of 3 biological replicates. Immunoblotting analysis showed similar levels of expression of Foxp1 and Ikaros in single and double transductions (Supplementary Figure S2). **B.** Schematic representation of the mouse *Gpr132* gene showing the positions of Regions A and B. The sequences of the two regions are shown underneath with the Foxp1 consensus binding sequences indicated by boxes. **C.** ChIP analysis of FLAG-Foxp1 binding to Regions A and B of the *Gpr132* gene. Results were expressed as % input precipitated in excess of background levels determined by amplification of two separate ‘gene-free’ regions of the genome (N1 and N2; see Methods). Values = mean ± SEM. n = 3 biological replicates.

Both Ikaros and Foxp1 have been shown to affect the cell cycle. Ikaros mediates exit of pre-B cells from the cell cycle [8] and Foxp1 is able to either increase or block proliferation depending on the cellular context [27–29]. This led us to consider that the interaction between the two proteins might influence cell cycle gene regulation. To address this question, we used a retroviral vector to overexpress Foxp1 in the B3 mouse pre-B cell line. Expression of the tagged Foxp1 was confirmed by western blotting (Supplementary Figure S2). A commercially available QPCR-based expression array (see Methods) was used to search for Foxp1 targets among genes that are known to be involved in cell cycle regulation. The results of the analysis showed that *Gpr132* was the most highly upregulated gene in the Foxp1-overexpressing cells, compared with empty vector-transduced controls (2.8-fold increase, Supplementary Table S1). *Gpr132* mRNA levels were further quantified by qRT-PCR analysis, which showed a mean 5-fold increase in transcription in the Foxp1 overexpressing cells (p<0.001, n = 3 biological replicates) (Figure 4A, Foxp1).

Since the interaction of Ikaros with the DNA binding domain might be expected to affect the action of Foxp1 on the *Gpr132* gene, we tested whether co-expression of Ikaros with Foxp1 affected *Gpr132* expression levels. Overexpression of Ikaros alone had no effect on *Gpr132* expression (Figure 4A, WT Ikaros), but co-expression of Ikaros with Foxp1 abolished the upregulation of *Gpr132* expression that was observed with Foxp1 only (Figure 4A, WT Ikaros +Foxp1). In contrast, co-expression with the IK6 isoform, which lacks the Foxp1 interaction domain, did not affect the Foxp1-mediated increase in *Gpr132* expression (Figure 4A, IK6 +Foxp1).

### Binding of Foxp1 to the Gpr132 gene is inhibited by wild-type Ikaros but not by IK6

To identify regions within or flanking the *Gpr132* gene that might be Foxp1 targets in B cells, ENCODE data for the gene and surrounding sequences obtained from adult (8 week-old) murine B-cells (https://www.encodeproject.org/) were mined for candidate enhancer elements. The search identified two regions of DNase I hypersensitivity located upstream and downstream from the promoter (designated as Regions A and B respectively). Analysis of the sequences of the two regions identified a Foxp1 consensus binding site at −1kb relative to the transcription start site (TSS) and four consensus sites at +4kb relative to the TSS (Figure 4B).

B3 pre-B cells were infected with a retroviral vector expressing FLAG-tagged Foxp1. Chromatin immunoprecipitation (ChIP) analysis with anti-FLAG antibody was used to assess binding of Foxp1 to Regions A and B. The ChIP analysis showed strong binding of Foxp1 relative to background in both regions. Co-transduction with a vector that expressed wild-type Ikaros reduced binding to near-background levels (Figure 4C). Importantly, transduction with a vector that expressed the IK6 isoform of Ikaros had no effect on binding of Foxp1 to Regions A or B. These results demonstrate that *Gpr132* expression is positively regulated by Foxp1 and that this regulation is antagonised by the interaction with Ikaros, but not by Ikaros isoforms that lack the DNA binding domain.

The human *GPR132* gene and its surrounding sequences were also searched for the presence of consensus FOXP1 binding sites. Two regions containing consensus sites were identified at approximately 3.5 kb upstream from the start site and 2 kb downstream in the first intron (Supplementary Figure S4A). We performed ChIP analysis of REH cells with anti-FOXP1 antibody, which showed binding of FOXP1 to both regions (Supplementary Figure S4B). These data therefore support the hypothesis that FOXP1 is directly involved in regulating expression of human *GPR132*, and suggest the existence of a functional homology between regulation of the mouse and human genes in pre-B cells.

### Ikaros antagonises cell cycle effects of Foxp1 and this effect is abolished by the IK6 deletion

The results described above indicate that Foxp1 enhances expression of the *Gpr132* gene, which is known to affect cell cycle progression [19, 30] and that Ikaros interacts directly with Foxp1 and antagonises its effect on *Gpr132* expression. To directly test whether cell cycle effects of Foxp1 expression are modulated by Ikaros, B3 cells were transduced with Foxp1- and Ikaros-expressing retroviral vectors, either singly or together. Propidium iodide staining and FACS analysis were used to measure the effect on cell cycle profiles.

**Figure 5:**
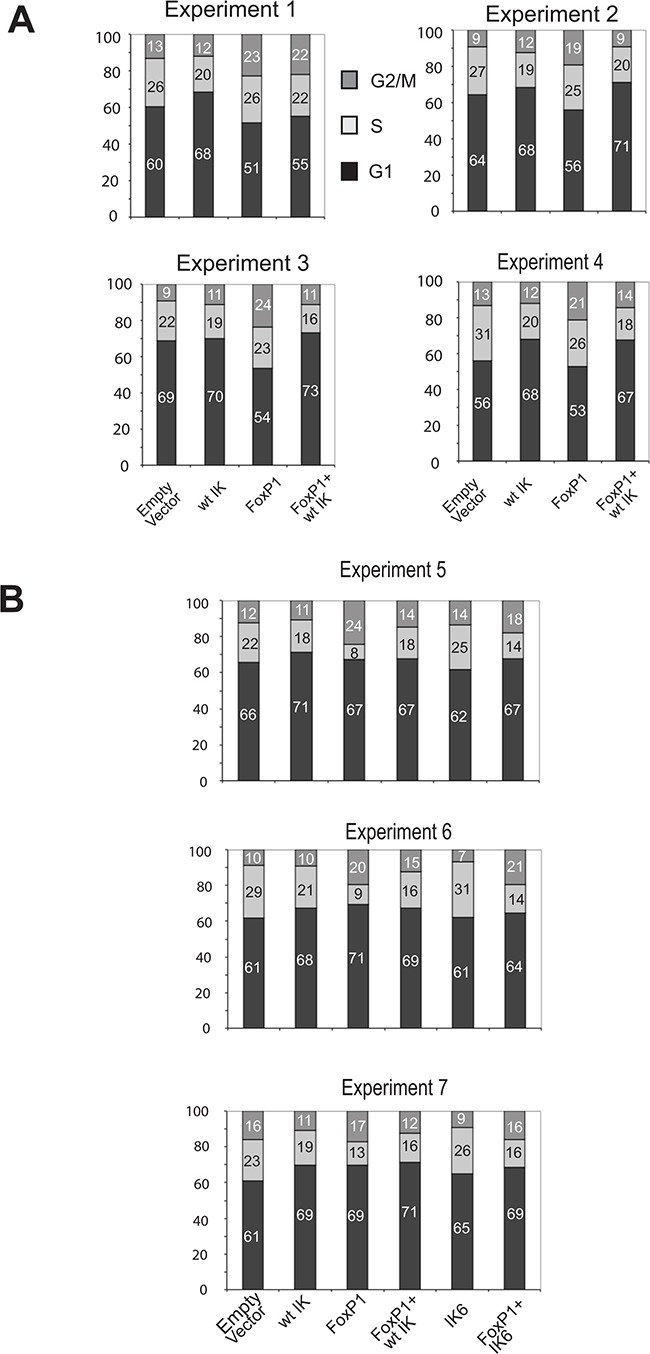
Cell cycle effects of Foxp1 are antagonised by wild-type Ikaros but not by IK6 **A.** Percentage of cells at different stages of the cell cycle analysed by PI staining and FACS. Data was obtained from 4 experiments where B3 pre-B cells were transduced with Ikaros and/or Foxp1 retroviral expression constructs. Individual percentage values (rounded to whole numbers) are shown within the histograms. Examples of the FACS profiles used to generate the cell cycle charts are shown in Figure S3. **B.** Percentage of cells at different stages of the cell cycle for 3 additional experiments where the effect of transducing cells with an IK6 expression vector on Foxp1-induced cell cycle changes was compared with the effect of expressing wild-type Ikaros.

The results of 7 biological replicate experiments are shown in Figure 5A and 5B. Overexpression of Foxp1 resulted in cell cycle arrest with a significant proportion of cells accumulating in G2/M and variable reduction of the number of S-phase cells and exit into G1. Co-expression of Ikaros and Foxp1 abolished the effect of Foxp1 on the cell cycle. In 3 of the 7 experiments, the effect of co-transducing IK6 and Foxp1 expression vectors was also measured (Figure 5B). In contrast to the effect of co-expressing wild-type Ikaros, co-expression of IK6 and Foxp1 did not prevent the increased G2/M arrest that resulted from the expression of Foxp1 alone (Figure 5B).

### IKZF1 deletion is associated with higher GPR132 expression in BCR-ABL negative B-ALL patients

**Figure 6:**
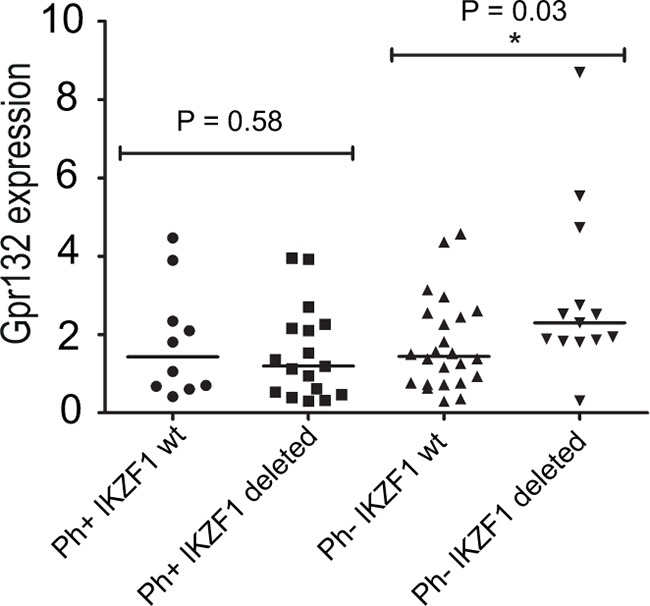
GPR132 levels in primary human B-ALL samples *GPR132* expression was quantified by qRT-PCR. Each point denotes the expression of *GPR132* in an individual sample relative to a *HPRT* housekeeping gene control. Ph+ = Philadelphia (BCR-ABL) positive. wt = Wild-type. Sample numbers in each group were as follows: Ph+ *IKZF1* wt, n= 10; Ph+ *IKZF1* Deleted, n = 15; Ph- *IKZF1* wt, n = 25; Ph- *IKZF1* Deleted, n = 15. The indicated p values are the results of statistical comparison of each patient group using a two-tailed unpaired t-test. Horizontal bars indicate median levels in each group.

The results described above suggest that the availability of Ikaros protein to interact with nuclear Foxp1 directly affects downstream gene regulation by Foxp1. This led us to speculate that mutations and deletions that reduce the level of WT Ikaros could result in aberrant regulation of *GPR132* expression in human B-ALL. To test this hypothesis directly, RNA was extracted from primary samples of human B-ALL at diagnosis and analysed for *GPR132* expression by qRT-PCR. The patient cohort included both BCR-ABL positive cases that have a high incidence of Ikaros deletions, and BCR-ABL negative cases that comprised both Ikaros WT and Ikaros-deleted samples (for details of deletions, see Supplementary Table S2). The analysis revealed a statistically significant (p<0.05) increase of 61% in levels of the *GPR132* transcript in Ikaros-deleted cases within the BCR-ABL negative group of patients, when compared with their Ikaros WT counterparts (Figure 6). In contrast, for patients in the BCR-ABL positive cohort, no significant difference in *GPR132* expression was observed between samples that did or did not have Ikaros deletions. Analysis of *FOXP1* expression levels in the same patient samples showed no evidence of increased *FOXP1* expression (Supplementary Figure S5A) and no detectable correlation between the expression levels of *FOXP1* and *GPR132* (Supplementary Figure S5B), indicating that the increased *GPR132* expression is not caused by increased expression of FOXP1.

## DISCUSSION

The results of this study reveal the existence of a novel interaction between Ikaros and Foxp1 that modulates the activating effect of Foxp1 on expression of the G protein-coupled receptor G2A, and potentially affects cell cycle progression in B lymphocytes. Our data additionally suggest that impairment of this interaction by the IK6 isoform alters G2A expression and could affect the cell cycle in B-ALL. The interaction does not depend on simultaneous binding of the two factors to DNA, but our results do not exclude the possibility that the factors could interact while binding to the same DNA regions in vivo and that this could have phenotypic effects depending on the context.

Ikaros and Foxp1 have important roles in B cell development, including the regulation of proliferation and cycling of lymphoid progenitors. Ikaros is required for progression to the pro- and pre-B cell stages and has a key role in cell cycle exit during the transition from the cycling large pre-B cell to the non-cycling small pre-B cell where light-chain rearrangement takes place. Foxp1 is required for efficient production of pre-B cells and is involved in regulating expression of the *RAG* genes. Foxp1 is also known to have anti-proliferative effects in several different cell types [27–29].

The observation that Ikaros and Foxp1 interact opens up an important new dimension to their respective functions. The forkhead factors are known to interact with a wide range of transcription factors and co-factors as a means for diversifying and modulating their functional effects during cell differentiation [31]. It is notable that the Ikaros family member Eos has been shown to interact with the forkhead protein Foxp3 in CD4+ regulatory T cells where it plays an important role in Foxp3-mediated silencing [32]. In an oncogenic context, physical interaction of FOXP3 with the transcription factor RUNX1 has been shown to regulate the expression of breast cancer related genes [33].

We have focused on the effect that the Ikaros-Foxp1 interaction has on the regulation of the *Gpr132* gene, which we have identified as a novel target for upregulation by Foxp1. Binding of Foxp1 to upstream and intronic sequences was observed at the mouse and human *Gpr132* genes. The fact that binding of Foxp1 to the mouse *Gpr13*2 gene is antagonised by expression of Ikaros suggests that the interaction of Foxp1 with Ikaros interferes with its ability to upregulate *Gpr132* expression. Increased levels of the G2A protein encoded by *Gpr132* have been shown to have complex cell cycle effects that include exit into either G2/M or G1, depending on cellular context and the action of specific phospholipid modulators of G2A function [34]. Interestingly, G2A expression is associated with cell cycle exit into G1 in keratinocytes [30], which is similar to the effect of Foxp1 on these cells. In our experiments, overexpression of Foxp1 resulted in cell cycle arrest, mainly in G2/M, and changes in the distribution of the different cell cycle stages. This pattern was abolished by co-expression with Ikaros, which resulted in a shift to a predominantly G1 arrest similar to the profile obtained with Ikaros alone. This result supports our hypothesis that expression of wild-type Ikaros is dominant over the cell cycle effects of Foxp1.

An important finding from our study is the observation that the IK6 deletion, which removes the Ikaros DNA binding domain and prevents the interaction with Foxp1, abolishes the effect of Ikaros expression on Foxp1 binding to the mouse *Gpr132* gene and on Foxp1-mediated enhancement of *Gpr132* expression. Cells that were co-transduced with Foxp1 and the IK6 deletion construct had a cell cycle profile similar to cells that were transduced with Foxp1 only. This was in contrast to the dominant effect of wild-type Ikaros over the cell cycle effects of Foxp1 and implies that the IK6 deletion is unable to antagonise the effects of Foxp1 on *Gpr132* expression and the cell cycle.

The hypothesis that reduced Ikaros protein levels upregulate *GPR132* expression was further supported by the finding that expression of the gene was significantly increased in Ph^−^ B-ALL patients with *IKZF1* deletions, compared with patients that have wild-type Ikaros. Intriguingly, the effects on *GPR132* expression were only observed in BCR-ABL negative samples, and no increase was found in patients that were positive for the BCR-ABL translocation. The *GPR132* gene product, G2A, has been shown to antagonise the oncogenic effects of BCR-ABL [21] raising the possibility that downregulation of *GPR132* expression is necessary for BCR-ABL transformation. This suggests that the presence of BCR-ABL downregulates *GPR132* expression, either directly, or through a downstream effector, overriding any effects that differences in FOXP1 binding to the *GPR132* gene might have. Conversely, absence of the BCR-ABL protein in BCR-ABL-negative B-ALL would allow overexpression of *GPR132* with the additional effects that this overexpression generates.

Current evidence indicates that BCR-ABL-negative B-ALL is generated by a complex spectrum of oncogenic mechanisms. A subset of BCR-ABL-negative cases have a gene expression profile that is similar to that of BCR-ABL-positive B-ALL and are described as Ph-like B-ALL [35]. However, these patients have a range of oncogenic lesions [36], including rearrangements involving *ABL1*, *ABL2*, *CRLF2*, *CSF1R*, *EPOR*, *JAK2*, *NTRK3*, *PDGFRB*, *PTK2B*, *TSLP*, or *TYK2* indicating that heterogeneous mechanisms are responsible for the phenotypes that are observed. The significant increase in *GPR132* expression that is associated with haploinsufficiency for *IKZF1* in these patients adds a potential mechanism for changes to the cell cycle that merits further investigation.

GPCRs are known to be overexpressed in a wide variety of malignancies, and are considered to be involved in cancer initiation, progression and metastasis (Reviewed in [37]. GPCRs are established common targets of drugs in clinical use, and GPCR antagonists constitute 30-40% of marketed drugs in humans [38]. Concerted efforts are therefore underway to develop pharmacological GPCR antagonists with anti-cancer activity. Our identification of increased *GPR132* levels in a subset of B-ALL adds to a growing body of evidence that GPCRs are aberrantly expressed in a spectrum of hematological malignancies. Evidence for GPCR dysregulation has been documented in acute myeloid leukemia [39], chronic lymphocytic leukemia [38, 40] and intestinal T-cell lymphoma [41]. Our results, together with the known association of cell cycle changes with poor prognosis [42–45] provide a rationale for investigation of G2A as a potential target in Ikaros-deleted B-ALL.

## MATERIALS AND METHODS

### Cell culture

Cells were cultured under the following conditions: WT murine pre-B: RPMI + 15% FCS, 2mM L-Glutamine, 50μM β-mercaptoethanol, 2ng/ml IL-7. 293T: DMEM + 10% FCS, 2mM L-Glutamine. REH and SUP-B15: RPMI + 20% FCS, 2mM L-Glutamine. B3 pre-B: IMDM+ 10% FCS, 2mM L-Glutamine, 50μM β-mercaptoethanol, 2ng/ml IL-7.

### Plasmid constructs

Ikaros and Foxp1 cDNAs were cloned into PCDNA3.1 vectors containing either a HA or double FLAG epitope tag. For GST-tagged proteins, cDNAs were inserted into pGEX-4T (GE lifesciences). Retroviral expression constructs were derived from the pMSCV-IRES-GFP plasmid.

### Preparation of nuclear extracts

Protein extracts were prepared as previously described [46, 47]. Cells were washed in PBS and initially resuspended in a low osmolarity buffer (10mM HEPES pH 7.9, 10mM KCl, 0.1mM EDTA and 0.1mM EGTA) and allowed to swell on ice for 15 minutes. After addition of NP40 (0.6%), the solution was mixed vigorously by vortexing, and subjected to centrifugation at 10,000 × g at 4°C for 30 seconds. The nuclear pellet was recovered and resuspended in a high osmolarity nuclear lysis buffer (20mM HEPES pH 7.9, 400mM NaCl, 1mM EDTA and 1mM EGTA) and incubated with shaking at 4°C for 30 minutes. Nuclear extracts were recovered in the supernatant following centrifugation at 10,000 × g at 4°C for 5 minutes.

### Co-immunoprecipitation

Following treatment with DNase I (Roche), protein extracts were incubated with the antibody-coupled G Dynabeads (Sigma) for 30 minutes at room temperature. Immunocomplexes were eluted by boiling the beads in 1x NuPAGE LDS Sample Buffer (Invitrogen) for 10 minutes at 70°C. Antibodies are listed in Supplementary Table S3. The anti-Ikaros and anti-Foxp1 antibodies used in the study recognise the human and mouse proteins.

### GST pulldown assay

Ikaros constructs (250 ng) were *in vitro* transcribed and translated with TNT T7 Quick Master Mix (Promega) and ^35^S methionine. For the GST pull-down assay, 15 μl of GSH–agarose beads and bacterial protein extracts containing either GST alone or GST–Foxp1 fusions were mixed and incubated at 4°C for 1 hour. Agarose beads were washed three times with 0.02 M HEPES–KOH pH 7.9, 0.1% NP-40, 0.15 M NaCl, 1 mM DTT and protease inhibitors. Immobilized GST proteins were then resuspended in 200 μl of the same buffer containing 1 μl of the *in vitro* translated proteins and incubated for 1 h at 4°C with rotation. The beads were washed three times with 1 ml of buffer. Proteins were eluted with 20 μl of loading buffer and boiled. Bound proteins were separated in a 10% SDS–polyacrylamide gel. Dried gels were analysed using a Fujifilm FLA-5100 scanner.

### Retroviral transduction of B3 cells

293T cells were co-transfected with pMIG retroviral vectors and pCL-Eco retrovirus packaging vector. Viral supernatant was collected at 48hrs and 72 hrs post-transfection. B3 cells were transduced with virus by spinoculation (700g × 60 minutes) in the presence of HEPES (10mM) and Polybrene (4μg/ml). Full details of the transduction protocol can be found in Supplementary Methods. Transduction efficiency was determined by FACS analysis of GFP expression 48 hours after transduction. For co-transductions, equal amounts of infective units of each virus were used.

### Quantitative reverse transcription PCR (qRT-PCR)

RNA was extracted using either an RNeasy Mini (cell lines) or Micro (primary samples) Kit (Qiagen), and retrotranscribed using Superscript III reverse transcriptase (Life Technologies). Changes in expression of cell-cycle-related genes in B3 cells transduced with a Foxp1-expressing retroviral vector were initially determined using the RT^2^ Profiler™ PCR Array Mouse Cell Cycle (Qiagen). Quantification of murine *Gpr132* expression in further experiments was obtained by normalizing to the housekeeping gene *Casc3*. *GPR132* expression in human samples was calculated by normalization to *HPRT*. Control and relative expression was calculated by the change-in-threshold (−ΔΔC_T_) method. Primer sequences are shown in Supplementary Table S4A (mouse) and S4b (human).

### Chromatin immunoprecipitation (ChIP)

ChIP was performed on formaldehyde-fixed B3 cells as described previously [48] using an anti-FLAG M2 antibody (Sigma-Aldrich). Primer sequences are shown in Supplementary Table S4c (mouse) and S4d (human). Binding to Regions A and B was compared with two genomic regions (designated N1 and N2), which to date have exhibited an absence of transcription factor binding in ChIP-sequencing data (N. Kunowska, personal communication). The positions of N1 and N2 are: N1: Chr17: 86,190,215-86,190,285; N2: Chr4: 55,591,773-55,591,842 (July 2007 (NCBI37/mm9) Assembly).

### Testing of IKZF1 deletion status

*IKZF1* deletion status was determined by analysis of a multiplex PCR reaction, as previously described [49]. Briefly, genomic DNA was extracted from patient samples using an Illustra DNA Extraction Kit BACC2 (GE Healthcare) and amplified using Amplitaq Gold® DNA Polymerase (Life Technologies) in a mix containing a series of fluorochrome-labelled primers (sequences available on request). Following amplification, PCR products were run on an ABI 3130 analyzer using a fragment size analysis program. Analyses of *IKZF1* deletions (indicated by the presence of truncated amplification products) were performed using GeneMapper software (Applied Biosystems).

## SUPPLEMENTARY MATERIALS FIGURES AND TABLES




