# Supplementary material for: Direct interaction of Ikaros and Foxp1 modulates expression of the G protein-coupled receptor G2A in B-lymphocytes and acute lymphoblastic leukemia
Source: Oncotarget. 2016 Aug 30;7(40):65923–36. doi: 10.18632/oncotarget.11688 (PMC5323203; doi:10.18632/oncotarget.11688)
Supplement: Supplementary file 1 [file oncotarget-07-65923-s001.pdf]

Direct interaction of Ikaros and Foxp1 modulates expression of the G protein-coupled receptor G2A in B-lymphocytes and acute lymphoblastic leukemia

SUPPLEMENTARY FIGURES AND TABLES

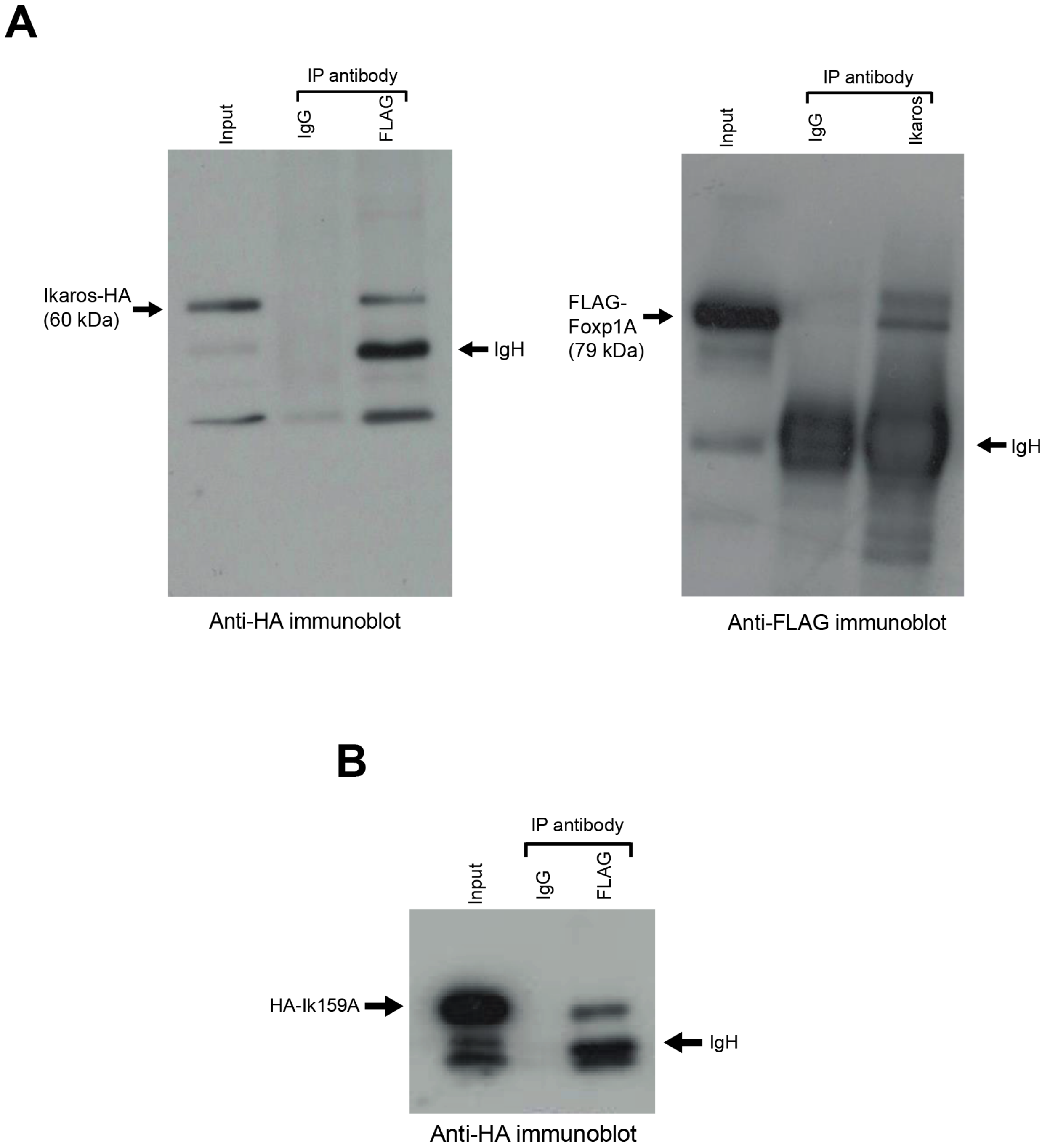

Supplementary Figure S1: The Ikaros-FoxP1 interaction is not dependent on DNA binding. **A.** 293T cells were co-transfected with FLAG-tagged Foxp1 and HA-tagged Ikaros constructs. Protein lysates prepared from these cells were treated with DNase. Left panel: Immunoprecipitation with anti-FLAG (Foxp1) followed by immunoblotting with anti-HA (Ikaros). Right panel: Immunoprecipitation with anti-Ikaros followed by immunoblotting with anti-FLAG (Foxp1). **B.** 293T cells were co-transfected with FLAG-tagged FoxP1 and HA-tagged Ikaros mutant 159A, which does not bind to DNA. Immunoprecipitation with anti-FLAG (FoxP1) followed by immunoblotting with anti-HA (Ikaros-159A) is shown.

A

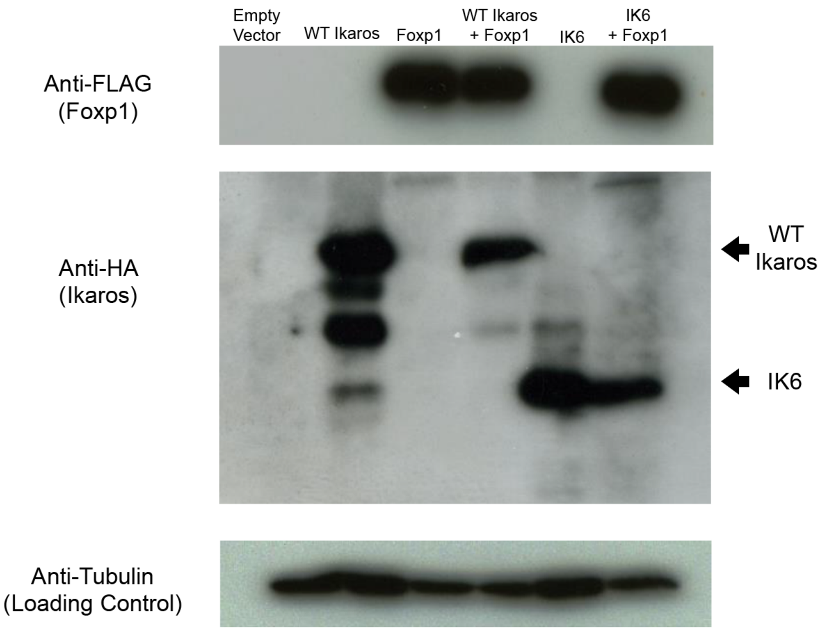

B

Infection	Relative HA-Ikaros Expression	Relative FLAG-Foxp1 Expression
WT Ikaros	1.00	
Foxp1		1.00
WT Ikaros + Foxp1	1.08	0.96
IK6	2.11	
IK6 + Foxp1	1.51	1.02

Supplementary Figure S2: Expression levels of FLAG-Foxp1 and of HA-Ikaros and IK6 in infected B3 cells. **A.** Immunoblotting analysis of the levels of ectopic protein expression for the B3 cell infections shown in Figure 2. The amounts of FLAG-Foxp1 and HA-Ikaros isoforms were determined using ImageQuant software and normalised to an anti-β-tubulin loading control. **B.** Relative molar ratios of ectopic protein expression calculated from the immunoblot shown in supplemental Figure 4a.

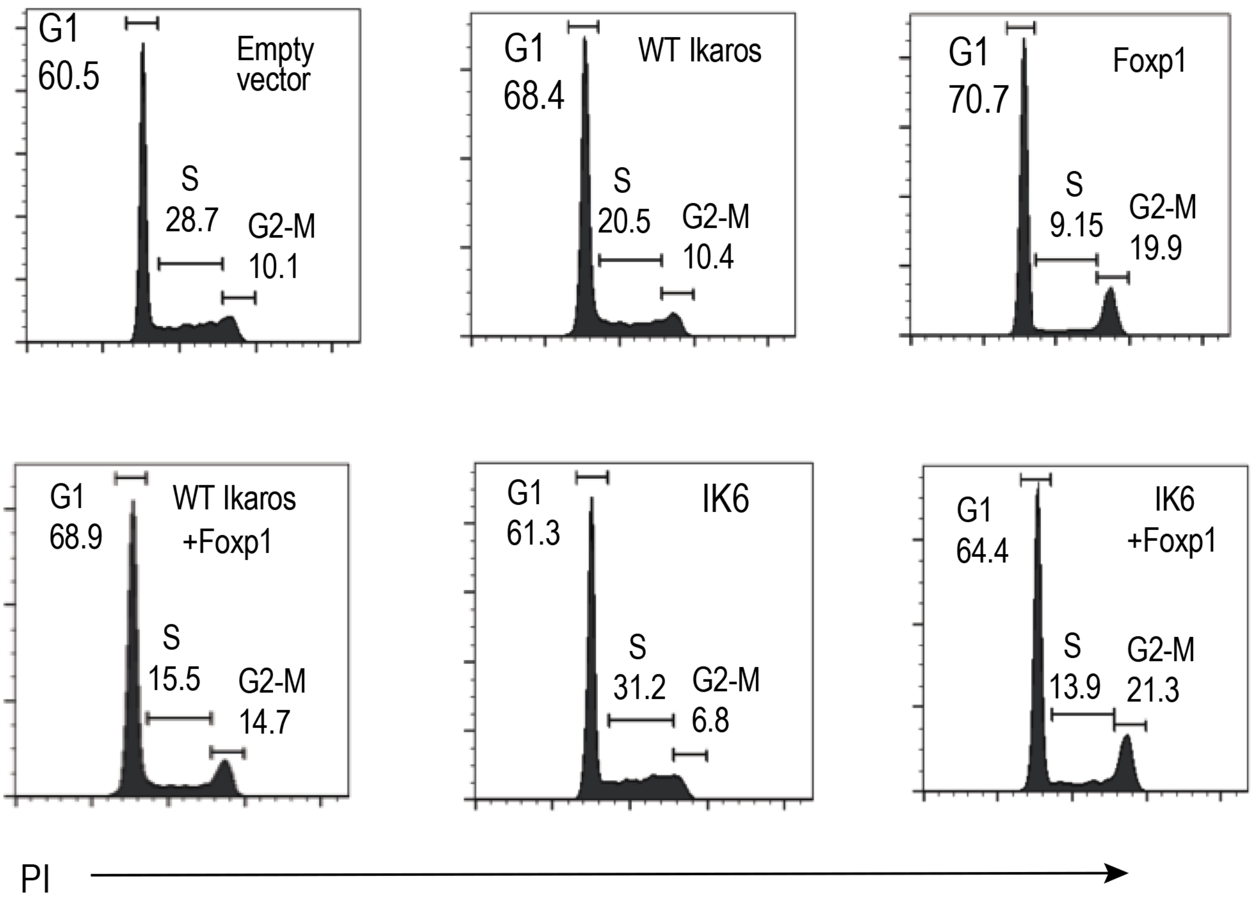

Supplementary Figure S3: Sample FACS plots from the data used to generate the cell cycle charts shown in Figure 4B, Experiment 6. Numbers represent the percentage of cells at each cell cycle stage.

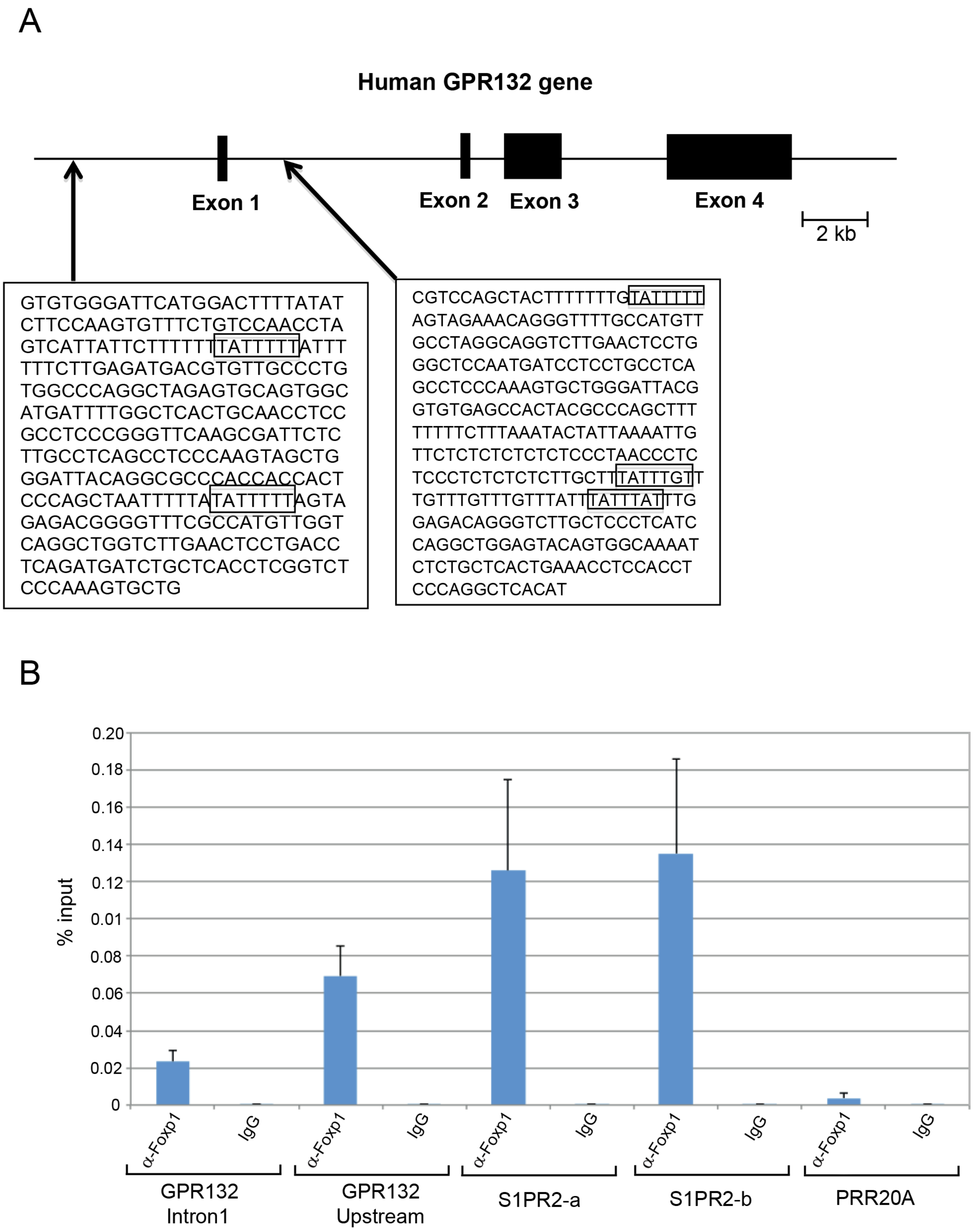

Supplementary Figure S4: ChIP analysis of binding of FOXP1 to the human *GPR132* gene. A. Sequences upstream from and within the first intron of the *GPR132* gene that contain consensus FOXP1 binding sites (indicated by open boxes). **B.** ChIP analysis in REH cells of the Intron 1 and upstream sequences shown in A using anti-FOXP1 antibody and non-specific control IgG. Two different regions (a and b) of the S1PR2 gene which have been shown to bind FOXP1in B-lineage cells [1] were used as positive control. A region of the PRR20A gene that was shown to be negative for FOXP1 binding [1] was used as a negative control. Values = mean ± SD. n = 2.

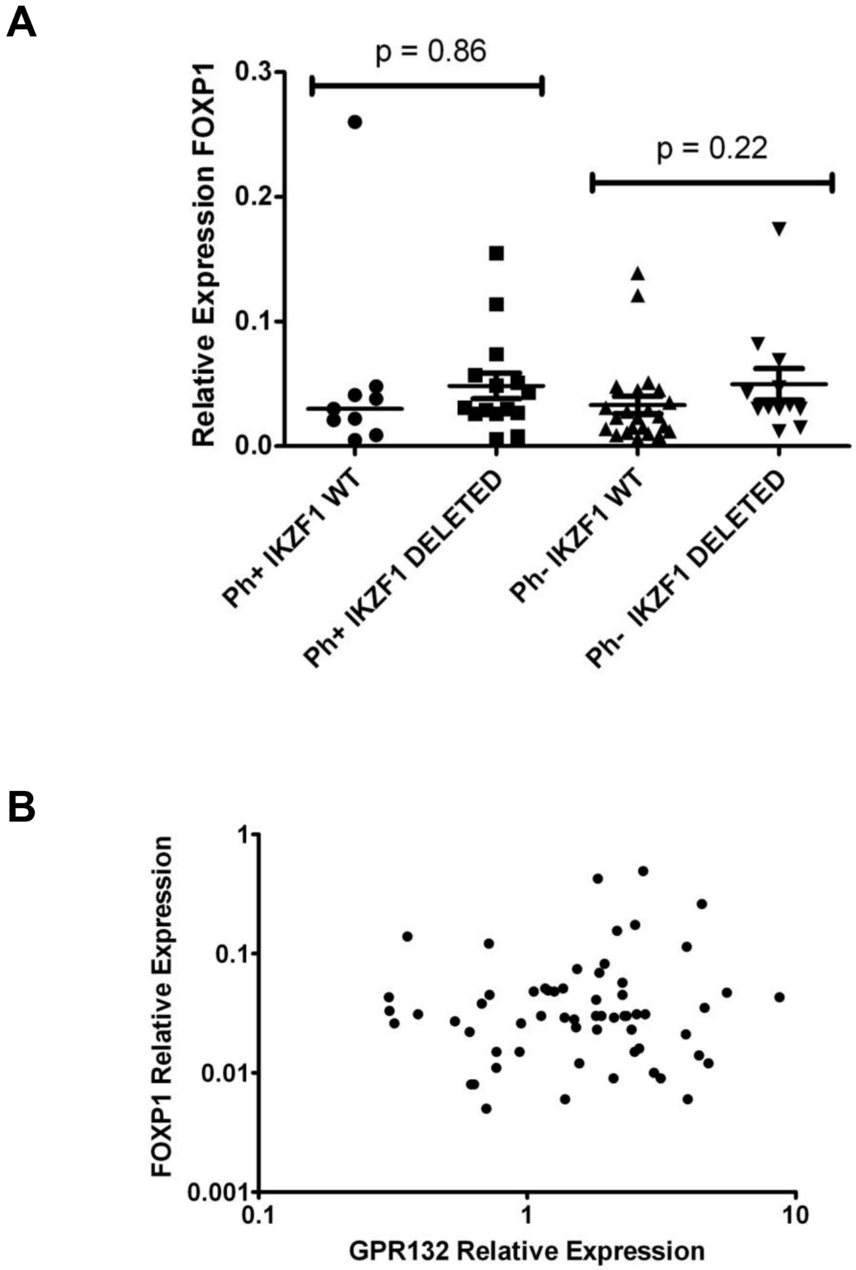

Supplementary Figure S5: Measurement of FOXP1 expression in human B-ALL patient samples A. Foxp1 levels in primary human B-ALL samples. FOXP1 expression was quantified by qRT-PCR. Each point denotes the expression of FOXP1 in an individual sample relative to a housekeeping gene control. Ph+ = Philadelphia (BCR-ABL) positive. wt = Wild-type. Sample numbers in each group were as follows: Ph+ IKZF1 wt, n= 9; Ph+ IKZF1 Deleted, n = 16; Ph- IKZF1 wt, n = 23; Ph- IKZF1 Deleted, n = 12. The indicated p values are the results of statistical comparison of each patient group using a two-tailed unpaired t-test. Horizontal bars indicate median levels in each group. **B.** Correlation plot of GPR132 and FOXP1 expression levels in primary human B-ALL samples.Each point represents the expression of FOXP1 and GPR132 for each patient, relative to a housekeeping gene control. There was no correlation between the levels of GPR132 and FOXP1 (Spearman r = 0.05).

Supplementary Table S1: Results of the the RT^2 Profiler™ PCR Array Mouse Cell Cycle (Qiagen). RT-QPCR was performed on RNA extracted from B3 cells transduced with either Foxp1 (Test) or an empty GFP vector (control). Fold changes and Up- or Down-regulation in Foxp1-transduced cells are shown in the indicated columns. Gpr132 expression is highlighted in blue.

See Supplementary File 1

Supplementary Table S2: Details of Ikaros deletions in BCR-ABL positive and BCR-ABL negative patients

Deletion	BCR-ABL POS	BCR-ABL NEG
Del Exon 2-7	6	4
Del Exon 4-7	11	8
Del Exon 4-8	2	1

In the BCR-ABL positive group, one patient had co-existent deletions of exons 2-7 and 4-7, and one patient had co-existent deletions of exons 2-7 and 4-8.

Supplementary Table S3: Antibodies

Antibody	Supplier
anti-FLAG M2 (monoclonal)	Sigma Aldrich (Cat F3165)
anti-HA (monoclonal)	Covance (Cat MMS-101P)
anti-Foxp1 (rabbit polyclonal)	Abcam (Cat ab16645)
anti-Ikaros (rabbit polyclonal raised against the C-terminal region of Ikaros [2])	Kindly provided by Dr. Steven Smale, UCLA.
Mouse IgG	Santa Cruz Biotechnologies (Cat sc-2025)
Rabbit IgG	Santa Cruz Biotechnologies (Cat sc-2345)
Goat anti-mouse IgG-HRP	Santa Cruz Biotechnologies (Cat sc-2005)
Goat anti-rabbit IgG-HRP	Santa Cruz Biotechnologies (Cat sc-2030)

Supplementary Table S4a: Primers used for QPCR analysis of gene expression (murine samples)

Name	Sequence
Gpr132 Primer Set A Forward	GCTACATGGCCGTGGTCTAT
Gpr132 Primer Set A Reverse	GGTGAAACGCAGGTAGTGGT
Gpr132 Primer Set B Forward	CGTCACCATCTTCCTGGTCT
Gpr132 Primer Set B Reverse	ACGTAGATGATGGGGTCAGC
Casc3 Forward	AGCTAACGATGCTGCTGATTC
Casc3 Reverse	TCCAAGTGCTTAGGGCCTTTT

Supplementary Table S4b: Primers used for QPCR analysis of gene expression (human samples)

Name	Sequence
GPR132 Primer Set A Forward	GGAGGGGTGCGAGGCTA
GPR132 Primer Set A Reverse	CATGTCATGCGTCTTGTCG
GPR132 Primer Set B Forward	GAGGCTAGCCACGCAGG
GPR132 Primer Set B Reverse	CGGGTTCCAATCTCAGTGTG
FOXP1 Forward	GTTGCAGTCCTGTGGCATTA
FOXP1 Reverse	TCACCTCAAAAGGTCACGTC
HPRT Forward	TGACACTGGCAAAACAATGCA
HPRT Reverse	GGTCCTTTTCACCAGCAAGCT

Supplementary Table S4c: Primers used for QPCR-ChIP (murine samples)

Name	Sequence
Gpr132 Region A; Primer Set 1, Forward	CGCAAAATCAGCATGAAGAA
Gpr132 Region A; Primer Set 1, Reverse	TGGGGCTCTGAAATATTGGT
Gpr132 Region A; Primer Set 2, Forward	TACAGACAACCAGGCACCAG
Gpr132 Region A; Primer Set 2, Reverse	TCATGCTGATTTTGCGTTTC
Gpr132 Region B; Primer Set 1, Forward	GAGGGCTGCTCAGGATGAT
Gpr132 Region B; Primer Set 1, Reverse	AGTGGTGGGTACCAGCAGTG
Gpr132 Region B; Primer Set 2, Forward	CTCACCCAACCCCTAACAGA
Gpr132 Region B; Primer Set 2, Reverse	CACGAACAGCTGCTTTCAGA
Background Region N1, Forward	CAGAGGACAGCTAGGCAGAGA
Background Region N1, Reverse	GAGCAGACTCATTAGGGCAAA
Background Region N2, Forward	TGATGACAGATCAAGCCAGGT
Background Region N2, Reverse	TTGTAACATGGGGTCGTG

Supplementary Table S4d: Primers used for QPCR-ChIP (human samples)

Name	Sequence
GPR132 Intron 1, Forward	GGATTACGGTGTGAGCCACT
GPR132 Intron 1, Reverse	AGCAAGACCCTGTCTCCAAA
GPR132 Upstream, Forward	CTTCATCGTAAGCGCAATCA
GPR132 Upstream, Reverse	ACATCCACCCATCAACACCT
S1PR2 Region A, Forward*	AGACTGTGTGTCACAGGCAA
S1PR2 Region A, Reverse*	TGCATTGCATTTCTGGGTGTATT
S1PR2 Region B, Forward*	CCGGGGACATCACCTTTGTC
S1PR2 Region B, Reverse*	TGGCTCAGCAATTTGCAGTC
PRR20A, Forward*	CTCCAGCAGTCGGCTTTC
PRR20A, Reverse*	GGAGGTGTCCACAGGTTCAC

* Described in [1]

REFERENCES

1. Flori M, Schmid CA, Sumrall ET, Tzankov A, Law CW, Robinson MD and Muller A. The hematopoietic oncoprotein FOXP1 promotes tumor cell survival in diffuse large B-cell lymphoma by repressing S1PR2 signaling. Blood. 2016; 127:1438-1448.

2. Hahm K, Ernst P, Lo K, Kim GS, Turck C and Smale ST. The lymphoid transcription factor LyF-1 is encoded by specific, alternatively spliced mRNAs derived from the Ikaros gene. MolCell Biol. 1994; 14:7111-7123.
